# Silencing of Diphthamide Synthesis 3 (Dph3) Reduces Metastasis of Murine Melanoma

**DOI:** 10.1371/journal.pone.0049988

**Published:** 2012-11-20

**Authors:** Lei Wang, Yu Shi, Peijun Ju, Rui Liu, Siok Ping Yeo, Yinyan Xia, Hamed Owlanj, Zhiwei Feng

**Affiliations:** 1 School of Biological Sciences, Nanyang Technological University, Singapore, Singapore; 2 Medical School, Xi’an Jiaotong University, Xi’an, China; University of South Alabama, United States of America

## Abstract

Melanoma is the most dangerous skin cancer due to its highly metastatic potential and resistance to chemotherapy. Currently, there is no effective treatment for melanoma once it is progressed to metastatic stage. Therefore, further study to elucidate the molecular mechanism underlying the metastasis of melanoma cells is urgently required for the improvement of melanoma treatment. In the present study, we found that diphthamide synthesis 3 (Dph3) is involved in the metastasis of B16F10 murine melanoma cells by insertional mutagenesis. We demonstrated that Dph3 disruption impairs the migration of B16F10 murine melanoma cells. The requirement of Dph3 in the migration of melanoma cells was further confirmed by gene silencing with siRNA in vitro. In corresponding to this result, overexpression of Dph3 significantly promoted the migratory ability of B16F10 and B16F0 melanoma cells. Moreover, down regulation of Dph3 expression in B16F10 melanoma cells strikingly inhibits their cellular invasion and metastasis in vivo. Finally, we found that Dph3 promotes melanoma migration and invasion through the AKT signaling pathway. To conclude, our findings suggest a novel mechanism underlying the metastasis of melanoma cells which might serve as a new intervention target for the treatment of melanoma.

## Introduction

Melanoma is a highly aggressive and frequently chemoresistant skin cancer caused by malignant transformation of melanocytes which are pigment-producing cells mainly found in the skin and eyes [1]. Although melanoma is only the third most common skin cancer in the world, it is the most deadly one and is responsible for 80% of deaths related to skin cancers [2]. At the early stage of development, most melanoma patients can be successfully treated by surgical resection of the tumor. However, currently, there is still no effective treatment for melanoma once it is progressed to metastatic phases [3], [4]. Metastasis is the spread of malignant tumor cells from a primary site to distant tissues and is the most life-threatening factor of cancer [5]. Metastasis is a multistep progress that can be envisioned a succession of cell biological changes, including cancer cells separating from original tumor, local invasion through surrounding tissues, intravasation into and transferring through the lymphatic vessels and blood stream, arresting in the parenchyma of distant tissues, formation of small nodules (micrometastasis), and finally, growth of micrometastatic lesions into macroscopic tumors (colonization) [6]. Although a variety of metastasis-promoting and metastasis suppressor genes, as well as microRNAs have been recently identified to be related to the metastasis of melanoma cells, the molecular mechanisms governing this process are still not completely understood and the treatment efficiency of metastatic melanoma has not been significantly improved over the past 50 years with a 5-year survival rate less than 5% [7]–[10]. Hence, further study to uncover related underlying mechanisms is urgently required to find new potential targets for the treatment of melanoma. In the present study, in order to identify genes involved in the metastasis of melanoma cells, we transfected B16F10 murine melanoma cells with a specifically designed retroviral vector pDisrup 8 [11]–[13] that can randomly disrupt genes in genome. The vector bearing cell clones were selected by blasticidin and then screened by wound healing to determine their migration potential. Cell clones with increased or decreased migratory potential were subjected to 3′RACE to identify genes involved in the metastasis of melanoma cells. As a result, we found that diphthamide synthesis 3 (Dph3) is involved in the cell migration of melanoma cells.

Dph3 is a small acidic protein with 82 amino acids, encoding a CSL zinc finger containing protein [14], [15]. It is evolutionally conserved and ubiquitously expressed in various mouse and human adult tissues. Dph3 is originally identified as a gene involved in the first step in the biosynthesis of diphthamide, a unique posttranslationally modified histidine residue occurs only in translation elongation factor 2 (eEF-2) [16]. Diphthamide is conserved from archaebacteria to eukaryotes [14]. Diphthamide biosynthesis is carried out by stepwise modifications to the His^715^ residues (His^699^ in yeast) of eEF-2 that requires the action of five proteins including Dph1-Dph5 [16]. Although the physiological roles of diphthamide are still not clear, the presentation of Dph1 to Dph5 in all eukaryotic organisms indicated that this modified histidine residue may have important biological functions. This notion is well supported by the fact that Dph1, also known as ovarian cancer-associated gene 1 protein (Ovca1), is a tumor suppressor gene that is frequently lost its heterozygosity in breast and ovarian tumors [17]–[19]. Further studies with Ovca1 heterozygous mice ascertained the significant roles of Ovca1 in cell proliferation, embryonic development and tumorigenesis [20], [21].

Except for its role in synthesis of diphthamide, Dph3 also plays an important role in embryonic development, as its genetic ablation causes embryonic lethality in mice. In addition, downregulation of Dph3 in Hela cells induces increased extracellular secretion of proteoglycans by association with DelGEF, a homologue of RanGEF [22]–[24]. The analogue of Dph3 in yeast, Kti11 (*Kluyveromyces lactis* killer toxin insensitive 11), has been found to be involved in regulating the sensitivity of yeast to zymocin [25], [26]. Furthermore, Kti11 has been reported to interact with varies proteins, such as core-Elongator (Elp1–Elp3), ribosome proteins, eEF2 and the diphthamide synthesis factors (Dph1 and Dph2), indicating that Dph3 may play roles in various biological processes [16], [27].

In the present work, we reported the identification of a novel role of Dph3 whose disruption impaired the metastasis of murine melanoma B16F 10 cells. By silencing and overexpression of Dph3, we further confirmed the role of Dph3 in the migration of melanoma cells in vitro. Moreover, reduction of Dph3 expression in melanoma B16F10 cells significantly impaired their metastasis in vivo. Further investigation demonstrated that AKT signaling pathway is involved in Dph3-promoted melanoma migration and invasion.

## Materials and Methods

### Cell Culture and Reagents

Murine melanoma B16F10, B16F0 cells, and NIH 3T3 cells were obtained from American Type Culture Collection (Manassas, VA, USA). Human melanoma cells were kindly provided by Dr Jean-Pierre Abastado (Laboratory of Tumor Immunology, Singapore Immunology Network, Singapore). Cells were cultured in Dulbecco’s Modified Eagle Medium with 10% fetal calf serum (FCS) (Gibco, Invitrogen, USA), and 1% Penicilin/Streptomycin mix (Gibco, Invitrogen, USA) and maintained at 37°C in a humidified atmosphere containing 5% CO_2_. Specific inhibitor for AKT (LY294002) was obtained from Calbiochem (USA). Lipofectamine 2000 was purchased from Invitrogen (USA).

### Plasmids and DNA Constructs

The pDisrup 8 vector was a gift from Dr. Han Jiahuai (Xiamen University, China). The short small interfering RNA (siRNA) was constructed with a sequence specifically targeted to mouse Dph3 gene: (5′-C AAG GAG TTA GTT AAA TGC -3′). Target and scrambled control oligonucleotides duplexes were cloned into pSilencer4.1-CMV vector (Ambion, USA) according to the manufacturer’s instructions. The Dph3 and its truncated colones were cloned into the sites of EcoRI and XhoI of pIRES2-EGFP vector (Clontech, USA) containing a Myc-tag with gene specific primers. The primers used were as follows: Dph3 (5′- CCG CTCGAG GCC ACC ATG GCG GTG TTT CAC GAC -3′and 5′- G GAATTC GCA TTT AAC TAA CTC CTTG-3′), Dph3 1-40aa (5′- CCG CTCGAG GCC ACC ATG GCG GTG TTT CAC GAC -3′ and 5′-G GAATTC TTCC AAA TCT TCC TTG GTG ATG-3′), Dph3 21-60aa (5′- CCG CTCGAG GCC ACC ATG ACA TAT TTC TAC CCT TGCC-3′ and 5′-G GAATTC GTC ATAAA TCA CTTTT ATAATG-3′), Dph3 41-82aa (5′- CCG CTCGAG GCC ACC ATG AAT GGA GAA GAT GTG GCC ACG-3′ and 5′- G GAATTC GCA TTT AAC TAA CTC CTTG-3′), Dph3 α–helix deletion (5′- CCG CTCGAG GCC ACC ATG GCG GTG TTT CAC GAC -3′, 5′- G GAATTC GCA TTT AAC TAA CTC CTTG-3′, 5′-GGA TAA CTT TGC CAT CAC CGG AGA AGA TGT GGC CACG-3′, 5′-CGT GGC CAC ATC TTC TCC GGT GAT GGC AAA GTT ATC C-3′). A constitutively active mutant D2AKT (T308D/S473D) plasmid was kindly provided by Dr. Takashi Tsuruo [28]. Transfection was performed with lipofectamine 2000 following the manufacture’s manual. For pDisrup 8 clone selections, cells were selected with Blasticidin S.HCl at 25 µg/ml (Invitrogen, USA).

### Western Blot

After washing with PBS (3.2 mM Na2HPO4, 0.5 mM KH2PO4, 1.3 mM KCl and 140 mM NaCl, pH 7.4) twice, cells were extracted with cold lysis buffer (20 mM Tris, 100 mM NaCl, 5 mM EDTA, 1 mM EGTA, 5 mM MgCl2, 1% Triton X-100, 2.5 mM sodium pyrophosphate, 1 mM b-glycerolphosphate, 1 mM Na3VO4, 1 mM PMSF, and Roche complete protease inhibitors) and centrifuged at 15,000 g for 15 min at 4°C. Protein concentration of the supernatants was determined with Bradford assay (Biorad, USA). 10–40 µg of samples was separated by electrophoresis on 8–16% SDS-PAGE and transferred to Polyvinylidene fluoride membrane (Millipore, USA). After blocking with 5% skimmed milk for 1 h, membranes were incubated with different specific primary antibodies in either 5% skimmed milk or 5% bovine serum albumin (BSA) (anti-GSK-3β, phospho-GSK-3β, β-Catenin, phospho-β-catenin, AKT, phospho-AKT, phospho-ERK1/2 from Cell Signaling Technology, anti-FAK from BD Bioscience, phospho-FAK from Upstate, anti-Flag from sigma). After washing with PBST for 30 min, the membranes were further incubated with corresponding HRP-conjugated secondary antibodies and developed with Pierce’s West Pico chemiluminescence substrate (Millipore, USA). All results were obtained from 3 independent experiments.

### Cell Attachment Assay

96-well tissue culture plates were coated with Collagen Type IV (Sigma Aldrich, USA) followed by washing with PBS before blocking with 0.5% BSA. 1×10^5^ melanoma cells were seeded onto the pre-coated 96-well plates and incubated for 15, 30, 60 and 120 min. After incubation, the unattached cells were removed and the plate was stained with 0.5% crystal violet in 20% methanol for 20 min at room temperature and then washed with tap water. Cell attachment was evaluated spectrophotometrically by dissolving the stain with 20% acetic acid and measured at a wavelength of 570 nm with Tecan (Männedorf, Switzerland).

### Wound Healing Assay

Wound healing assay was performed as previously described [29]. Briefly, B16F10 and B16F0 murine melanoma cells were seeded in 60 mm dishes and cultured at 37°C overnight to produce a confluent monolayer. After starvation in serum-free medium for 24 hours, a wound was created by scratching the monolayer with a 200 µl yellow sterile pipette tip. The wounded monolayer was then washed twice to remove cell debris and incubated with fresh normal medium. The area of cell-free scratch was photographed at 0 h, 12 h and 24 h after scratching respectively. The wound healing effect was determined by measuring the percentage of the remaining cell-free area compared with the area of the initial wound.

### In vitro Invasion and Migration Assay

Invasion of melanoma cells was determined by BD BioCoat™ Matrigel™ Invasion Chamber (BD Biosciences, USA) assay in vitro according to the manufacturer’s instructions. In brief, 1 × 10^5^ cells with 500 µl in serum-free medium were added into the upper chamber and 750 µl of NIH-3T3 fibroblast conditioned medium was added into the lower chamber, serving as chemo-attractant. After incubation in humidified tissue culture incubator, 37°C, 5% CO_2_ atmosphere for 24 h, the non-invasive cells in the upper surface of the membrane were removed by “scrubbing” with cotton tipped swab and the invasive cells migrating to the lower surface of the membrane were fixed and stained with 0.5% crystal violet for 30 minutes. Cell counting was then carried out by photographing the membrane through the microscope. 20 random fields under microscope at 20X magnification are taken. The migration assay was performed with the same strategy, just that the chamber membrane was not coated with matrigel while assessing cell motility.

### MMP-9 Activity Assay

The activity of MMP-9 was determined by QuickZyme Mouse MMP-9 activity assay (QucikZyme BioSciences, Netherlands) according to the manufacturer’s instructions. Briefly, after transfection for 48 h, cells were washed with fresh medium and replaced with serum-free medium. After additional 24 h, the medium was collected and centrifuged at 10000 g for 10 min. Respective supernatant was added to the 96-well strip coated with MMP-9 antibody and incubated at 4°C overnight. After washing with wash buffer for 4 times, 50 µl assay buffer was added into the well, followed by adding 50 µl detection reagent. After incubation at 37°C for 1 h, OD405 was measured with Tecan (Männedorf, Switzerland).

### Animals and Experimental Metastasis Assay

Female C57BL/6J mice at 6–8 weeks old (15–20 g) were purchased from the laboratory Animal Center of NUS (Singapore). Mice were maintained at dark/light cycles of 12 h duration with food and water available *ad libitum*. 12 animals were randomly divided into two experiment groups. For experimental metastasis analysis, the mice were injected at the lateral tail vein with (5×10^5^) B16F10 cells carrying control or Dph3 siRNA plasmids. Mice were sacrificed 2 weeks after inoculation and all organs were examined for the presence of macroscopic metastases. Lung and liver metastatic nodules were determined under a dissecting microscope. Animal handling and experimental procedures were approved by the Institutional Animal Care and Use Committee (IACUC) of Nanyang Technological University (ARF SBS/NIE-A0075).

### Real-time PCR

Total RNA was isolated using TRIzol according to the manufacturer’s instructions (Invitrogen, USA) and the concentration of total RNA was detected by spectrophotometry at OD260. Reverse transcription (RT) was carried out using superscript III reverse transcriptase (Invitrogen, USA) as described in the manufacturer’s manual. The real-time PCR was performed on ABI Prism 7500 Sequence detection system (Applied Biosystems, CA) with the KAPA SYBR® qPCR Kit (KAPA Biosystems, USA) according to the manufacturer’s instructions. The conditions for real-time PCR amplification were as follows: 2 min at 50°C, 1 min at 95°C, 40 cycles at 95°C for 3 s and 60°C for 1 min, followed by melting curve analysis at the end of each run from 60°C to 95°C. The primers used were as follows: Dph3 (Forward: 5′- TTG CCA TCA CCA AGG AAG ATTT-3′, Reverse: 5′-GTG CTG GGA CTG TTT CTC CAC -3′), MMP-9 (Forward: 5′-CTG GAC AGC CAG ACA CTA AAG -3′, Reverse: 5′- CTC GCG GCA AGT CTT CAG AG -3′), β-actin (Forward: 5′-GCT CTT TTC CAG CCT TCCTT-3′, Reverse: 5′-TGA TCC ACA TCT GCT GGAAG-3′). The target mRNA level of control cells normalized to the level of β-actin mRNA, was defined as 1. Results were obtained from three independent experiments.

### Statistical Analysis

For quantitative PCR analyses, results were obtained from triplicate experiments on all the samples and data from all trials were averaged. Numerical results were analyzed using independent mean T-test and expressed in mean ± standard error (SE). Statistical analysis was performed using post hoc testing using Bonferroni’s method. Differences were considered statistically significant at p<0.05.

## Results

### Identification of a Novel Role of Dph3 in Melanoma Metastasis

The murine melanoma B16F10 cell line is a widely used model to study the metastasis of melanoma for its high metastatic potential after intravenous injections [30], [31]. To identify genes involved in melanoma metastasis, we transfected melanoma B16F10 cells with pDisrup 8 vector to randomly produce insertions into the genomic DNA, followed by selection with blasticidin (25 µg/ml) to obtain mutated cell clones. The migratory ability of the selected mutant cell clones was then determined by wound healing assay. Finally, cell clones with increased or decreased migration potential were further analyzed by the RT-PCR and 3′ RACE to identify the genes disrupted by pDisrup 8 vector. With this strategy, several candidate genes were identified, including a gene named Dph3 and this candidate was designated as Dph3^mut^ which exhibited decreased motility potential.

**Figure 1:**
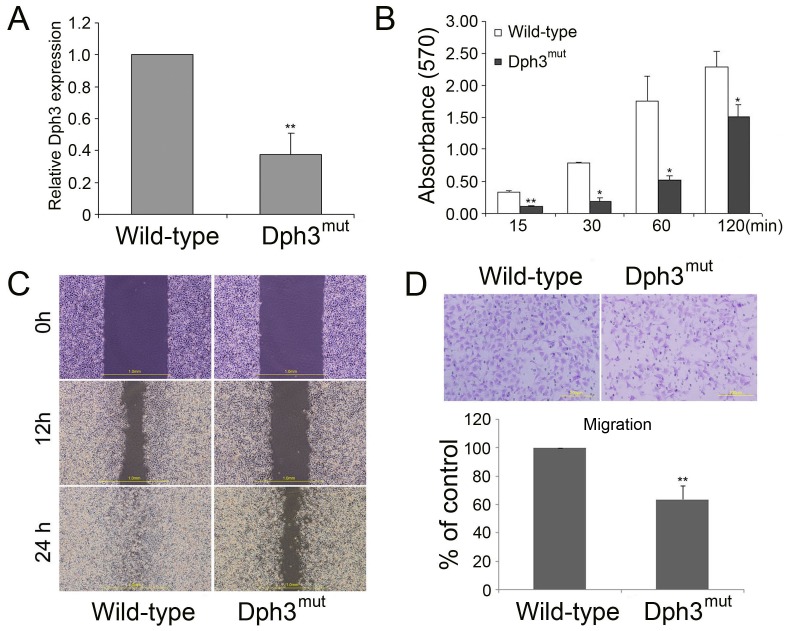
Identification of a novel role of Dph3 in the metastasis of melanoma B16F10 cells. A, Dph3 expression in Dph3^mut^ cells was analyzed by real-time PCR. B, a total of 1×10^5^ cells were seeded into pre-treated 96-well plates for the indicated times. Attached cells were stained with crystal violet and measured spectrophotometrically. Results are expressed as absorbance values. C, wound healing of control and Dph3^mut^ cells was performed and representative pictures of the wound distance were taken at each time point as indicated. Scale bars: 1 mm. D, the cell motility was evaluated by transwell assays. Representative pictures were taken after staining with crystal violet. Scale bars: 200 µm. Data are collected from three independent experiments and are average ±S.E. values. *p<0.05, **p<0.01, compared to wild type cells.

To verify whether the gene identified by this method was indeed disrupted in melanoma B16F10 cells, real-time PCR was carried out to determine the gene expression of Dph3. As shown in Figure 1A, the expression of Dph3 was greatly reduced in this cell clone compared to the control cells. Cellular invasion, a characteristic of metastatic tumors, involves cell attachment to extracellular matrix (ECM), ECM degradation and cell migration [32], [33]. To determine if loss function of Dph3 affects cell attachment and migration, cell attachment experiment was performed. Compared to control cells, disruption of Dph3 significantly reduced cell adhesion to substratum at different attaching period of time (Fig. 1B). Then we performed wound healing and transwell assays to evaluate the cell motility. As shown in Figure 1C, 12 h after scratching, the area of wound recovered by the migration of Dph3^mut^ cells was not significant and only less than half of that for control ones. 24 h later, wild type cells had almost closed up the wound, but not Dph3^mut^ cells. Consistently, there were less Dph3^mut^ cells that migrated across the membrane of the Boyden chamber compared to the wide type cells (Fig. 1D). In summary, disruption of Dph3 led to reduced cell adhesion and significantly impaired the migration of B16F10 cells.

**Figure 2:**
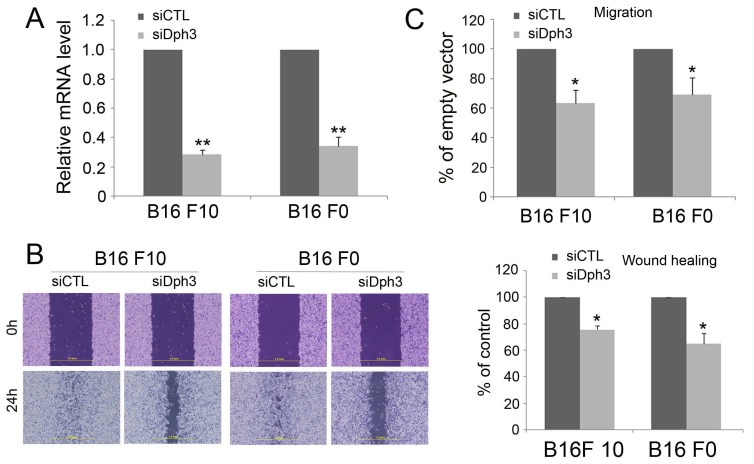
Dph3 silencing decreases the migration of melanoma B16F10 and B16F0 cells.

### Confirmation of the Role of Dph3 in Melanoma Migration by Gene Silencing and Overexpression

To ascertain the gene identified was indeed responsible for reduced migration in B16F10 cells, we investigated whether reduced migration of melanoma cells could be reproduced by gene silencing with siRNA. To perform this experiment, we first silenced the expression of Dph3 in melanoma B16F10 and B16F0 cells with a siRNA-incorporated plasmid targeting a specific site of mouse *Dph3* mRNA. As shown in Figure 2A, the expression of Dph3 in cells transfected with siRNA plasmid (siDph3) was significantly decreased compared with the cells transfected with scrambled siRNA (siCTL). Transfected cells were then subjected to wound healing and transwell assays to evaluate their migratory potential. Both B16F10 and B16F0 cells transfected with a control siRNA were able to close a wound by 24 h. However, the wound inflicted on cells transfected with Dph3 siRNA had not yet closed up at this time (Fig. 2B). Consistently, results from transwell assay also showed that the cell number of siDph3 cells moved across the membrane was much fewer than the siCTL cells (Fig. 2C). Taken together, these results indicated that silencing of Dph3 could reproduce the effect of Dph3 disruption by pDisrup 8 plasmid and drastically reduced the cell motility of melanoma cells.

**Figure 3:**
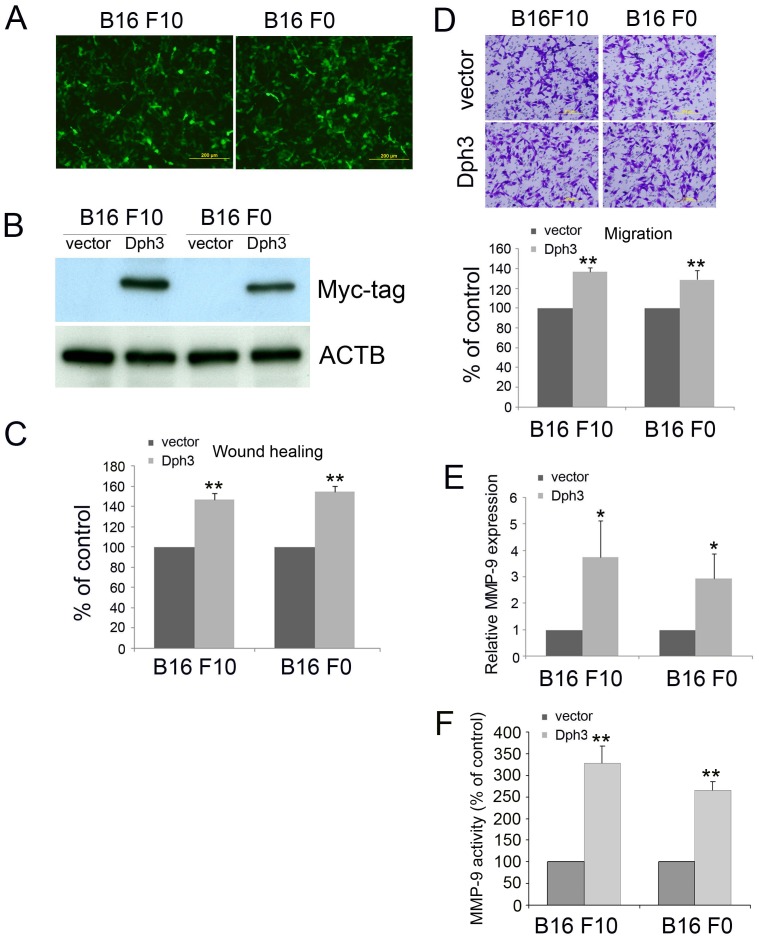
Dph3 overexpression promotes the migration of melanoma B16F10 and B16F0 cells. Dph3 was cloned into pIRES2-EGFP vector and transfected into melanoma B16F10 and B16F0 cells. The cells transfected with an empty vector were used as control. The transfection efficiency was evaluated by the expression of green fluorescence protein (GFP) (A) and the expression of Dph3 was determined western blot with Myc-tag antibody (B). Scale bars: 200 µm. β-actin was used as a loading control. The cell motility was determined by wound healing assay 18 h post scratching and the percentage of wound closure was quantified (C). D, the cell motility was evaluated by transwell assays. Scale bars: 200 µm. E, real-time PCR was carried out to evaluate the mRNA expression level of MMP-9 in control and Dph3 overexpression cells. F, activity of MMP-9 in control and Dph3 overexpression cells was determined by MMP-9 activity assay. Data are from three independent experiments and are average ±S.E. values. *p<0.05, **p<0.01, compared to control cells.

To determine whether Dph3 is widely expressed in melanoma cells, we examined the expression of Dph3 in various human melanoma cells (M102, SK-28, and 888-mel). As expected, we found that Dph3 is highly expressed in examined human melanoma cells (data not shown), especially in M102 cell line. Furthermore, in order to determine the cellular specific effect of Dph3 on tumor metastasis, we transfected human colon cancer cell HCT116, human ovary cancer cell A2780 and human skin cancer cell A431 with siDph3 plasmid and siCTL plasmid, followed by examination of the migration of these cell lines with transwell assay or wound healing. As shown in figure S1, siDph3 only affects the migration of A431 cells and has no effect on the migration of HCT116 and A2780 cells. These results suggested that Dph3 mainly affects the migration of some skin cancer cells.

**Figure 4:**
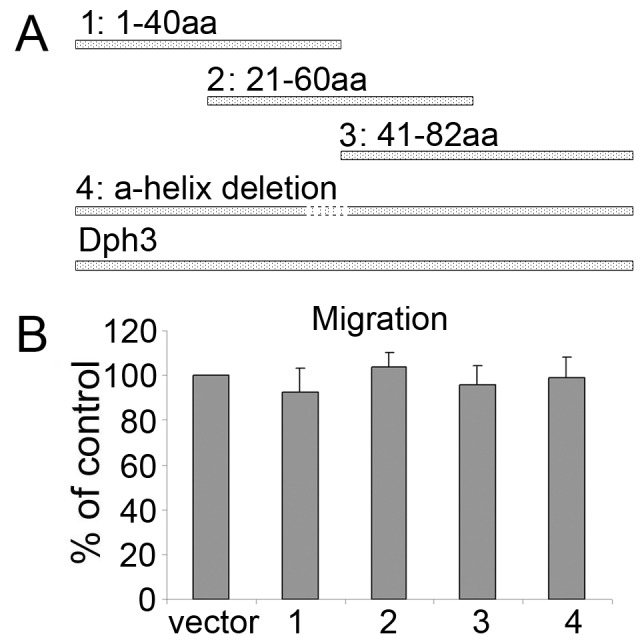
Full length of Dph3 was required for the metastasis of murine melanoma B16F10 cells. A, Schematic presentation of a mutant with a predicted 6 amino acids helix deletion between 36–41 of the whole protein sequence and 3 truncated mutants of Dph3. B, the cell motility was evaluated by transwell assays after transfection with different mutants.

**Figure 5:**
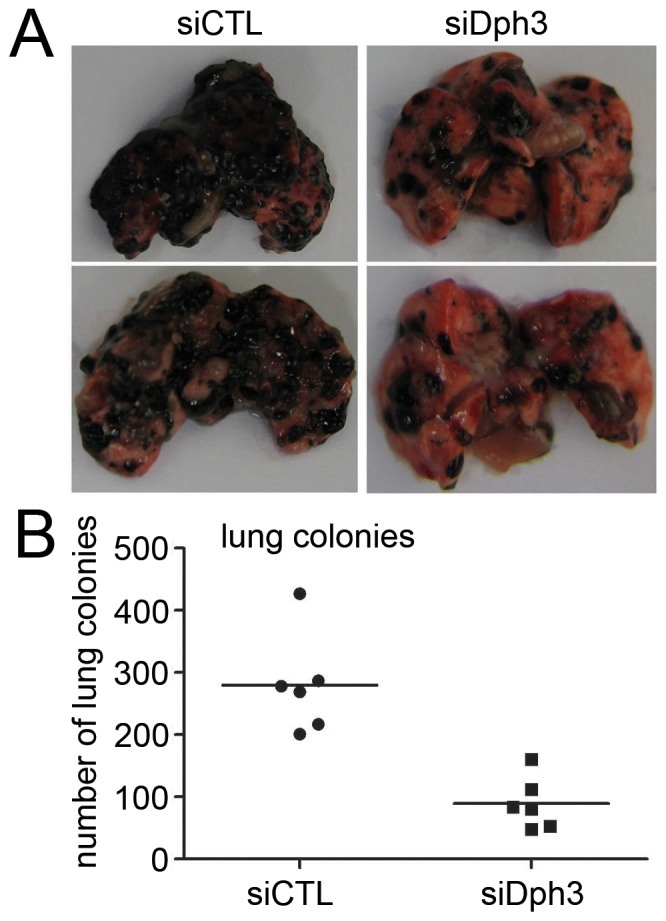
The metastasis of mouse melanoma B16 F10 was impaired by Dph3 silencing in vivo. siCTL and siDph3 cells were injected into the lateral vein of C57BL/6J mice respectively. A, representative pictures of lungs from mice were taken after 2 weeks of injection with B16F10 control cells or with Dph3 KD cells. B, numbers of lung metastasis were quantified and showed by each data point, **p<0.01, compared to control cells.

To further confirm the role of Dph3 in melanoma motility, we cloned Dph3 into pIRES-EGFP vector and transfected it into melanoma B16F10 and F0 cells. The transfection efficiency was confirmed by the expression of green fluorescence protein (GFP) (Fig 3A). Due to lack of effective antibody against Dph3, we further confirmed the overexpression of Dph3 by western blot with a Myc-tag antibody (Fig 3B). The migration of Dph3 overexpressed cells was then examined by wound healing assay and migration assay. As shown in Figure 3C, the wound gaps of cells which were stably expressing Dph3 gene healed faster than control cells. In accordance with this result, there were more Dph3 overexpressed cells migrated across the membrane (Fig 3D). Matrix metalloproteinases (MMP), which are capable of degrading the various structural components of the ECM, play a critical role in tumor invasion and metastasis and the up-regulation of matrix metalloproteinase (MMP)-9 has been considered as one of the markers for the metastasis of melanoma cells [34], [35]. To determine if Dph3 overexpression affects the transcription level of MMP-9, we then tested the effect of Dph3 overexpression on the expression of MMP-9 by real-time PCR. As expected, the mRNA expression of MMP-9 was significantly up-regulated after Dph3 overexpression (Fig. 3E). Consistent with this result, the activity of MMP-9 was also markedly increased with the overexpression of Dph3 (Fig. 3F). Collectively, these results suggest that Dph3 promotes motility of melanoma cells.

To determine the functional motif of Dph3 required for the metastasis of melanoma cells, a mutant with a predicted 6 amino acids helix deletion and 3 truncated mutants were constructed and transfected into melanoma B16F10 cells (Fig. 4A). The migration of cells transfected with truncated Dph3 clones was then examined by transwell migration assay. As shown in Figure 4B, all truncated mutants had no effects on the migration of melanoma B16F10 cells, indicating that the full length of Dph3 is responsible for its role in melanoma migration.

### Dph3 Knockdown Reduces the Metastasis of Mouse Melanoma B16 F10 in vivo

To further investigate the role of Dph3 in the metastasis of melanoma cells in vivo, an experimental metastasis assay was performed. Control and Dph3 knock down cells were injected into the lateral tail vein of C57BL/6J mice. 2 weeks post inoculation, animals were sacrificed and all the major organs were checked for the generation of tumor metastasis. The tumor metastasis was mainly observed in the lungs as previously reported [36]. We found that injection of B16F10 control cells resulted in the formation of numerous lung colonies (median, 280; range, 201–427) whereas silencing of Dph3 significantly suppressed pulmonary metastasis and only generated one third of lung colonies (median, 90; range, 48–160, p<0.01) (Fig 5B). In addition, B16F10 control cells produced nodules that occupied a higher percentage of the total lung area, while metastatic nodules of siDph3 cells generated discrete black foci (Fig 5A). These results implied that Dph3 silencing indeed perturbed the metastasis of melanoma cells not only in vitro but also in vivo.

**Figure 6:**
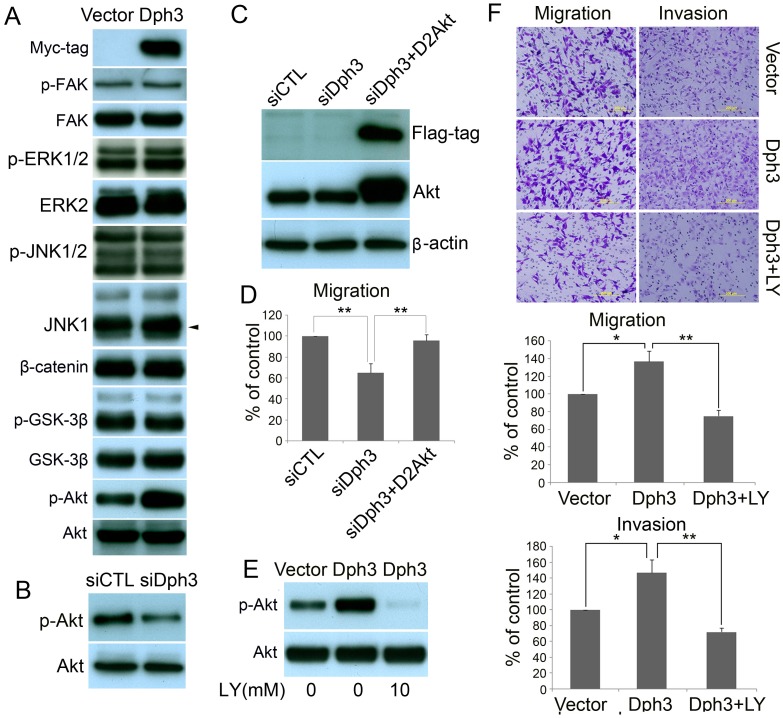
Dph3 facilities the invasion and migration of melanoma cells via AKT pathway. A, western blot shows that the phosphorylation of AKT at Ser-473 was elevated in cells transfected with pIRES-EGFP-Dph3. AKT was used as a loading control. B, the phosphorylation of AKT at Ser-473 was perturbed in cells transfected with Dph3 siRNA plasmid. AKT was used as a loading control. C, expression of D2AKT was confirmed by western blot with antibody against Flag-tag and AKT, and β-actin was used as loading control. D, transwell assays were performed to determine the motility of cells cotransfected with Dph3 silencing plasmid and D2AKT plasmid. Columns are data collected from three independent experiments. E, in the presence of LY294002 (20 µM), cells were incubated for 1 h, protein extracts were analyzed by western blot with antibodies against phosphorylated AKT (S473) or AKT. F, in the presence of LY294002 (20 µM), transwell assays (left panel) and Matrigel invasion assays (right panel) were conducted to evaluate the cell motility and invasiveness after transfection. Representative pictures were taken after staining with crystal violet. Columns are data collected from three independent experiments and are average ±S.E. values. *p<0.05, **p<0.01, compared to control cells.

### AKT Pathway is Involved in Dph3-mediated B16F10 Migration and Invasion

To determine the signaling pathways which are involved in Dph3-mediated melanoma cell migration and invasion, multiple potential signaling pathways related to migration and invasion of cancer cells were screened. As shown in Figure 6A, only the basal level of AKT activation was found to be significantly upregulated in cells overexpressing Dph3. In contrast, no obvious difference could be observed for many other signaling pathways, such as FAK, JNK, ERK and Wnt (β-catenin and GSK-3β). Consistently, when Dph3 was silenced by siRNA in melanoma cells, AKT activation was also down regulated (Fig. 6B). In combination, these results strongly suggest that Dph3 facilitates the activation of AKT signaling pathway in B16F19 murine melanoma cells. To confirm the role of AKT in Dph3-mediated cell migration, constitutively active form of AKT (D2AKT) was introduced into Dph3-silenced cells and the expression of D2AKT was confirmed by western blot with anti-Flag and anti-AKT antibody (Fig. 6C). As expected, active AKT largely restored the impaired migration in Dph3-silenced cells (Fig. 6D). Furthermore, LY292004, a PI3K/AKT specific inhibitor was also employed to dissect the role of PI3K/AKT signaling in cell migration and invasion. As shown in Fig. 5F, Dph3-mediated phosphorylation of AKT was completely blocked (Fig. 6E) by LY294002. As a result, Dph3-promoted migration and invasion in B16F10 cells were also abolished by LY294002, as shown by the transwell migration and invasion assays (Fig. 6F). To conclude, these data indicate that AKT signaling is involved in Dph3 promoted metastasis of melanoma cells.

## Discussion

Malignant melanoma is the skin cancer with the highest risk of death for its highly metastatic potential [37]. Both incidence and mortality rate continue to climb in recent years [38]. However, there is currently no effective treatment for metastatic melanoma partly due to the complicated mechanism underlying its metastasis. In the present study, we identified a novel role for Dph3 in the metastasis of murine melanoma cells. We found that Dph3 promotes the metastasis of murine melanoma cells in vitro and in vivo through the AKT signaling pathway. Our results may provide a new target for intervention in the melanoma treatment and may improve the future treatment of melanoma.

Dph3 is originally identified as a gene involved in the biosynthesis of diphthamide. Diphthamide is conserved in all eukaryotes and archaea and is important for ribosomal protein synthesis, especially for translation fidelity in preventing -1 frameshift mutation [39]. Dph1-4 and Dph5 catalyzed the first and second step of diphthamide biosynthesis, respectively [40]. Although diphthamide is essential for cellular translation, abolishing its synthesis is not embryonic lethal [14]. However, it has been recently reported that Dph3 knock out in mice leads to embryonic lethality [24]. Moreover, recent studies had shown that loss of Kti11, the homologue of Dph3 in yeast, can also cause growth defects [26]. Hence, it had been proposed that Dph3 may play additional roles, other than diphthamide synthesis in the cells. So far, no investigation has been carried out on the involvement of Dph3 in cancer. In this work, we firstly reported a novel role for Dph3 in the metastasis of melanoma cells. We found that the metastasis of B16F10 murine melanoma cells was significantly inhibited, when the Dph3 gene was disrupted by insertional mutagenesis. Further investigation with gene silencing of Dph3 showed that the metastasis of melanoma cells was significantly decreased as revealed by the wound healing assay and migration assay and this effect is specific to skin cancer cells (Fig S1). In contrast, the overexpression of Dph3 in murine B16F10 and B16F0 melanoma cells greatly enhanced the migration of melanoma cells. More convincingly, Dph3 silencing markedly impaired the lung metastasis of murine melanoma B16F10 cells in vivo. All these data presented that Dph3 is involved in the metastasis of melanoma cells. Interestingly, compared to the pro-metastatic effect of Dph3, Dph1, which deletion in mouse leads to embryonic lethality as Dph3, was reported as a tumor suppressor gene for breast and ovarian cancer [20], [22]. In Dph1 heterozygous mice, cancer can spontaneously develop at 80–102 weeks of age [22]. The reason why Dph1 and Dph3 function so differently in tumor cells needs to be further investigated.

The underlying molecular mechanism for Dph3-regulated melanoma metastasis is identified to be related to AKT signaling pathway. Our results showed that overexpression of Dph3 in B16F10 murine melanoma cells induces upregulation of AKT phosphorylation and Dph3 silencing leads to reduced AKT phosphorylation. Furthermore, restored AKT activity by an active form AKT plasmid could rescue the impaired migration of B16F10 cells induced by Dph3 silencing. In the presence of LY294002, a specific inhibitor of AKT signaling pathway, Dph3 overexpression-promoted migration and invasion were significantly inhibited as revealed by the transwell migration and invasion assay. The pro-metastatic potential of AKT pathway can be supported by the involvement of upregulation of phosphorylated AKT in severely dysplastic nevi and metastatic melanomas compared with normal or mildly dysplastic nevi [41]. Recently, AKT was reported to be a downstream effecter of UCH-L1 in regulating the tumor-cell invasion [42]. Moreover, activation of AKT pathway is also required in human breast cancer cells and ovarian cancer cells to promote cellular invasion and metastasis [43]. However, the detail mechanisms underlying Dph3 regulating the metastasis of melanoma cells is till not clear. We speculated that the mechanism leading to this specificity involves the ability of Dph3 to associate with guanine nucleotide exchange factor DelGEF, a homologure of RanGEF [23]. Guanine nucleotide exchange factors regulate the activity of small GTPase by promoting the release of GDP or GMP and the binding of GTP to GTPase [44]. Dph3 directly interacts with DelGEF, which is a protein previously reported to regulate extracellular secretion of proteoglycans in HeLa cells by association with Sec 5, a component of Sec6/8 complex [22], [23]. The Sec6/8 complex has been demonstrated to interact with several GTPase such as Rho1 [45] and RalA [46]. Dph3, possibly in combination with DelGEF, regulates the binding of nucleotides to GTPase. The small GTPase Ras has been reported to activate PI3K by directly interacting with the catalytic subunit of type I PI3Ks, leading to activation of the lipid kinase by inducing its translocation and conformational changes [47]. It is possible that Dph3 might regulate the activation of AKT by the small GTPase Ras though association with guanine nucleotide exchange factors.

Due to the important roles of Dph3 in the metastasis of melanoma B16F10 cells, it may serve as an attractive target for molecular targeting cancer therapy. In the further work, it is worthwhile to elucidate the precise roles of Dph3 in regulating the AKT signaling thus mediating the metastasis in melanoma cells.

## Supporting Information

Figure S1 Dph3 silencing decreases the migration of human skin cancer A431 cells, not human ovary cancer A2780 cells and human colon cancer HCT116. pSilence4.1-CMV vectors carrying siDph3 or siCTL were transfected into human skin cancer A431 cells, human ovary cancer A2780 cells and human colon cancer HCT116 separately. The cell motility of A2780 (A) or HCT116 (B) was evaluated by transwell assay. C, the cell motility of A431 cells was determined by wound healing assay and representative pictures of the wound distance were taken at 0 and 18 h post scratching as indicated. The percentage of wound closure was quantified (right). Data are from three repeated experiments and are average ±S.E. values. *p<0.05, **p<0.01, compared to control cells.
(TIF)
