# Supplementary figures and images for: Silencing of Diphthamide Synthesis 3 (Dph3) Reduces Metastasis of Murine Melanoma
Source: PLoS One. 2012 Nov 20;7(11):e49988. doi: 10.1371/journal.pone.0049988 (PMC3502187; doi:10.1371/journal.pone.0049988)

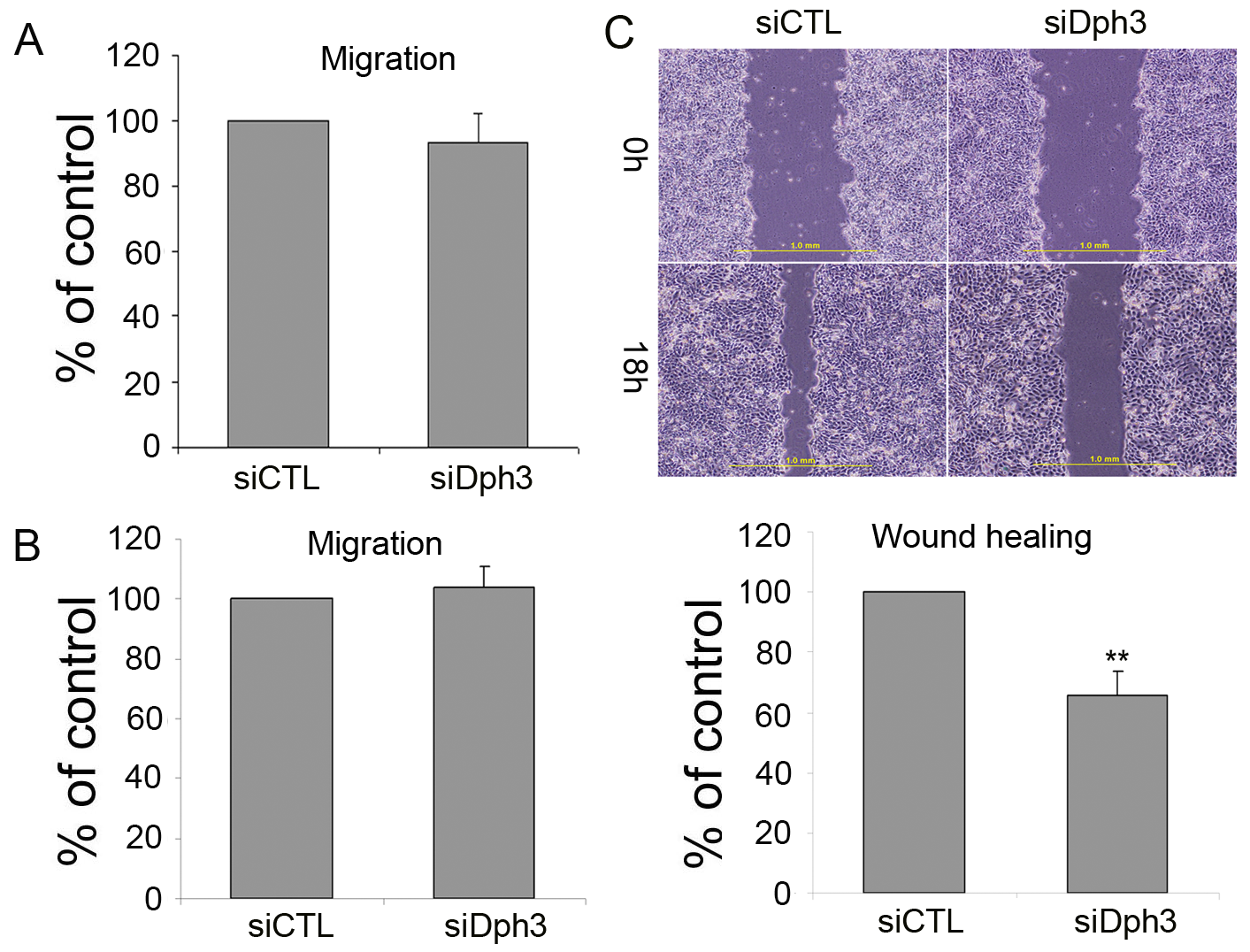

Supplement: Figure S1 — Dph3 silencing decreases the migration of human skin cancer A431 cells, not human ovary cancer A2780 cells and human colon cancer HCT116. pSilence4.1-CMV vectors carrying siDph3 or siCTL were transfected into human skin cancer A431 cells, human ovary cancer A2780 cells and human colon cancer HCT116 separately. The cell motility of A2780 (A) or HCT116 (B) was evaluated by transwell assay. C, the cell motility of A431 cells was determined by wound healing assay and representative pictures of the wound distance were taken at 0 and 18 h post scratching as indicated. The percentage of wound closure was quantified (right). Data are from three repeated experiments and are average ±S.E. values. *p<0.05, **p<0.01, compared to control cells. (TIF) [file pone.0049988.s001.tif]
